# A population-based study of the sex-specific associations between apolipoprotein B and incidence of atrial fibrillation

**DOI:** 10.1186/s12944-026-02905-6

**Published:** 2026-02-23

**Authors:** Yingying Wei, Lintao Wang, Chao Zhang, Biao Xu, Linda S. Johnson, Gunnar Engström, Xue Bao

**Affiliations:** 1 Department of Cardiology, Cardiovascular Disease Center, Jiangsu Key Laboratory for Cardiovascular Information and Health Engineering Medicine, Nanjing Drum Tower Hospital, Nanjing Drum Tower Hospital Clinical College, Nanjing University of Chinese Medicine, No. 321 Zhongshan Road, Nanjing, 210008 China; 2 Department of Clinical Sciences, Lund University, Malmö, Sweden

**Keywords:** Apolipoprotein B, Atrial fibrillation, Cohort studies, Sex differences

## Abstract

**Background:**

Apolipoprotein B (apoB) is a well-known risk factor for atherosclerosis. However, studies examining its relation to atrial fibrillation (AF) have produced conflicting results and suggested possible sex-specific differences. This study investigated the sex-specific associations between serum apoB concentrations and incident AF and offer insight into the inconsistencies in previous research.

**Methods:**

A prospective analysis of 26,803 participants without pre-existing AF was performed using data from the Malmö Diet and Cancer Study. Sex-specific associations between apoB and AF were assessed using multivariable Cox proportional hazards models. To ensure the robustness of the results, several sensitivity analyses, such as restricted cubic spline modeling, competing risks regression, alternative adjustment strategies, subgroup analyses, follow-up time restrictions, and multiple imputation for missing data, were conducted.

**Results:**

For median follow-up periods of 21.2 and 24.8 years in men and women, respectively, 2,768 and 2,968 incident cases of AF were recorded, respectively. Among women, unadjusted models showed a strong positive association between apoB and AF, with the highest versus lowest quartile showing a hazard ratio (HR) of 1.65 (95% confidence interval [CI] 1.49–1.84; *P* for trend < 0.0001). The association became non-significant after age adjustment (*P* for trend = 0.09), and was reversed after multivariable adjustment (HR 0.77, 95% CI 0.69–0.86; *P* for trend < 0.0001). Sensitivity analyses consistently supported a significant linear inverse association in women. No significant link between apoB and AF was detected in the male population. Most sensitivity analyses were similarly null except for the restricted cubic spline analysis which suggested a borderline non-linear association (*P* for effect = 0.04, *P* for nonlinearity = 0.05). *Post-hoc* analysis suggested an inverse association at lower apoB concentrations (*P* = 0.0012) (≤ 100 mg/dL: HR per standard deviation 0.90, 95% CI 0.85–0.96).

**Conclusions:**

Results show sex-specific observational links between apoB concentrations and risk of AF. In women, higher apoB levels were linearly inversely associated with AF, whereas in men, the association was borderline non-linear, with inverse effects seen mainly at lower apoB concentrations. These sex differences in AF susceptibility may partly reflect underlying atrial electrophysiological variations and hormonal influences, though whether these factors directly mediate the apoB-AF association remains speculative.

**Supplementary Information:**

The online version contains supplementary material available at 10.1186/s12944-026-02905-6.

## Introduction

As the most prevalent cardiac arrhythmia in the elderly, AF constitutes a major contributor to morbidity and mortality [1]. Although the precise mechanisms remain incompletely elucidated, the pathogenesis of AF involves a complex interaction of atrial structural and electrical remodeling [1]. Dyslipidemia, especially high low-density lipoprotein (LDL), is a known risk factor for atherosclerosis and coronary heart disease [2], which are associated with a higher risk of AF [3].

Apolipoprotein B (apoB) is the major structural protein of LDL. LDL particles enter the arterial wall through endocytosis and in susceptible areas, apoB is bound to arterial proteoglycans, and LDL can remain in the subcutaneous space [4]. The deposition of LDL and other apoB-containing particles in the arterial intima is now recognized as an important initiating factor in atherogenesis [5].

Given the close association of atherosclerosis with AF [3], one would expect to see a positive association with AF risk for apoB levels. However, in contrast to the well-established link between apoB and atherosclerosis, a “cholesterol paradox” has been noted in AF in which traditional lipid indices, such as LDL, are inversely associated with AF risk [6, 7]. Whether apoB, an important causal atherogenic particle, shows a similar paradoxical relationship or positive association with AF is unclear. Cross-sectional studies have reported an inverse link between apoB and AF [8, 9]. In contrast, longitudinal studies have yielded inconsistent results [10–12]: while an inverse association was found in a female cohort [10], no significant association was found in a male cohort [11] or in mixed-sex populations [12]. These discrepancies indicate the existence of sex-specific associations between serum apoB and AF, which may be affected by sex hormones or sex-specific differences in body fat distribution [7]. Nevertheless, sex-stratified analyses of the apoB-AF relationship are still scarce and limit a clear understanding of the observed inconsistencies [10–12].

To address this gap, the present large population-based cohort study set out to explore sex-specific relations between serum apoB and incident AF, so as to gain insights to resolve conflicting findings from previous research.

## Methods

### Participants

**Fig. 1:**
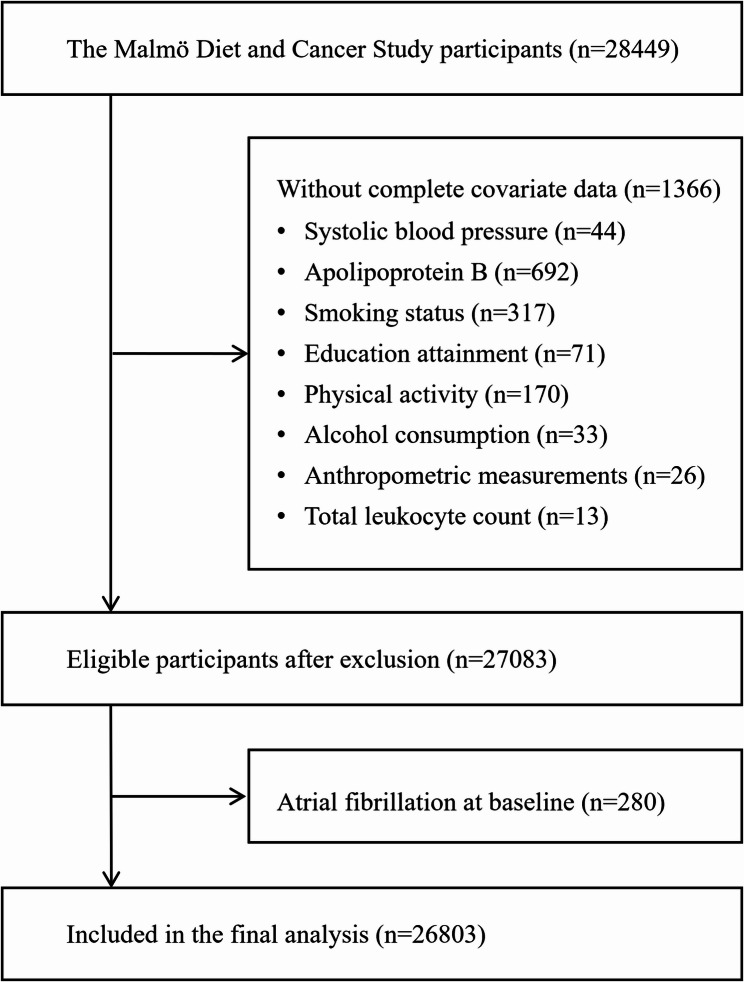
Study population flow chart

The Malmö Diet and Cancer Study (MDCS) is a population-based prospective study that was started in 1991, in which 11,246 men and 17,203 women living in the city of Malmö, Sweden were recruited [13]. Baseline assessments (1991–1996) included standardized physical examinations, fasting blood sample collection and validated self-reported lifestyle questionnaires, as described previously [13]. Of the 28,449 participants originally recruited, 27,083 (95.2%) had complete covariate data. After excluding 280 individuals with pre-existing AF, and retaining those with other cardiometabolic or atherosclerotic conditions, the final analytical cohort was 26,803 (10,453 men and 16,350 women), aged 45–73 years. The participant selection flow is depicted in Fig. 1. This study adhered to the Declaration of Helsinki (1964, revised 2013), approved by the Lund Regional Ethics Committee (LU 51/90; 2023 − 00503), and obtained written informed consent from all participants before enrolling.

### Baseline measurements of apoB and covariates

Blood samples were collected at baseline, immediately centrifuged and kept under cryopreserved conditions after rigorous, standardized protocols for handling, storing and quality control of samples, as described in the protocols for the operations of the Malmö Biological Bank [13, 14]. Serum apoB concentrations were quantified in cryopreserved specimens (-80 °C) by immunonephelometric analysis (Siemens BNII system, Siemens Healthcare, San Juan Capistrano, CA, USA) at Quest Diagnostics with an inter-assay coefficient of variation of < 4%. Blood pressure and anthropometric parameters were measured by calibrated sphygmomanometers and stadiometers according to standardized procedures [13]. Demographic and lifestyle variables were obtained through validated questionnaires, such as smoking status (current [regular/occasional] versus non-smoker), alcohol intake (high intake: > 40 g/day for men; > 30 g/day for women), medication use, educational level (post-secondary education [> 12 years] versus not), and physical activity (quartile-stratified according to an 18-domain composite score). Additional information included age at menopause, history of thyroid-related conditions, such as goiter, thyroid hormone replacement therapy, or antithyroid medication use. Diabetes mellitus was identified through self-report, hypoglycemic medication use, or confirmation by the Swedish Hospital Registers for inpatients and outpatients. All biochemical analyses followed standardized clinical chemistry protocols, as previously described [13, 14].

### Ascertainment of outcomes

Participants were followed from baseline until the first occurrence of incident AF, death, emigration from Sweden or end of follow-up on December 31, 2020. Outcome data were extracted from Swedish national registries kept by the National Board of Health and Welfare, namely the Hospital Discharge Register (inpatient diagnoses), the Hospital-based Outpatient Register (outpatient diagnoses), and the Cause of Death Register. These registries have good population coverage with no missing data resulting from registry linkage. AF cases were identified by international classification of diseases (ICD) codes in three revisions: ICD-8 427.92 (pre-1986), ICD-9 427D (1987–1996), and ICD-10 I48 (post-1996) [15], which covers both AF and atrial flutter because of their similar pathophysiological mechanisms [16]. For the validation, 100 randomly selected cases from the cohort were reviewed based on electrocardiograms (available for 98% of cases) and patient records [15]. Of these, 95% were confirmed as definite AF, 3% as non-AF and 2% without electrocardiograms were classed as probable AF.

### Statistical analysis

Participants were stratified by sex-specific apoB quartiles. Their baseline characteristics were presented as mean ± standard deviation (SD) for normally distributed continuous variables, median (interquartile range) for skewed variables, and frequency (percentage) for categorical variables. Comparisons according to apoB quartiles and between sexes were performed, with linear regression used for continuous variables and logistic regression used for categorical variables. Pearson or Spearman correlation analyses were conducted to investigate the correlations between apoB and traditional cardiometabolic risk factors. Associations between apoB levels, analyzed both by quartiles and by 1 SD increase, and incident AF were examined using Cox proportional hazards models. Follow-up time was adopted as the time scale. Interaction effects of sex and apoB on AF risk were formally tested and *post-hoc* analyses were performed separately by sex. The proportional hazards assumption was checked via Schoenfeld residuals and log(-log(survival)) plots. Sequential adjustment was applied: unadjusted (Model 1); age-adjusted (Model 2); further adjusted for smoking, alcohol intake, education, and physical activity (Model 3); fully adjusted for Model 3 covariates and body mass index, systolic blood pressure, total leukocyte count, diabetes status, and use of antihypertensive or lipid-lowering medications (Model 4); and Model 5, further adjusted for age at menopause, for women. Covariates were chosen according to known AF risk factors [17] and clinical importance. *Post-hoc* exploratory analyses of baseline data were performed to determine differences in these covariates according to apoB levels. Variance inflation factors (VIFs) were computed to examine possible multicollinearity between covariates. Effect modification was assessed using multiplicative interaction terms between apoB and covariates, and stratified analyses were conducted when significant interactions were found. To estimate the possible effect of unmeasured confounding, E-values were calculated. To test the robustness of the findings, several sensitivity analyses were carried out. Non-linear associations between apoB and AF were tested using restricted cubic spline (RCS) analyses with 4 knots located at the quintiles of apoB concentrations and included in adjusted Cox models. Competing risks regression was performed with Fine-Gray sub-distribution hazards to control for non-AF mortality, which allows for sub-distribution hazard ratios (HRs) and competing events as informative censoring [18, 19]. Cumulative incidence functions for AF with death as a competing event were plotted by apoB quartiles. Given the close relationship between AF and atherosclerotic diseases [20], a sensitivity analysis excluded the participants with baseline stroke or coronary events. In this analysis, incident stroke or coronary events before AF during follow-up, in addition to non-AF deaths, were considered as competing events. To control for the possible effect of thyroid dysfunction on the risk of AF [21] and lipid levels [22], further adjustment was made for a history of thyroid-related conditions (including goiter or levothyroxine treatment). Several age-related sensitivity analyses were performed because of the high correlation between age and AF [17]: (1) using age rather than follow-up duration as time scale; (2) conducting age-stratified analyses (cut-off: 60 years); and (3) using RCS as a time-dependent variable. To address possible reverse causation, cases of AF during the first 5 years of follow-up were excluded in a separate sensitivity analysis. Long-term mortality effects were investigated by limiting follow-up to 15 years. In a sub-cohort for which C-reactive protein (CRP) data were available (*n* = 4,884), analyses were further adjusted for CRP levels. Pearson correlations were also determined between apoB and other lipid parameters (LDL, high-density lipoprotein [HDL], total cholesterol, triglycerides) in a random sample of participants (*n* = 5,003). Given the close biological relationship between apoB and LDL, a discordance analysis was carried out by cross-classifying the participants using sex-specific median values for both markers. To assess the possibility of bias resulting from missing data, multiple imputation by chained equations was performed using 50 imputed data sets. All statistical analyses were 2-tailed, with *P* < 0.05 considered statistically significant, and performed using version 9.3 of SAS for Windows (SAS Institute, Cary, NC, USA).

## Results

### Baseline characteristics

The comparison of baseline characteristics between participants excluded and included in the analysis (after excluding those with baseline AF) is shown in Table S1. Within the included cohort, baseline apoB concentrations, which were normally distributed, were higher in men than women (*P* < 0.0001; Table 1). Most of the cardiometabolic risk factors demonstrated progressive increases between sex-specific apoB quartiles (*P* < 0.0001), except for high alcohol consumption (*P* = 0.02) and advanced education (*P* < 0.0001) which showed inverse trends. Overall, women had lower levels of most risk factors than men (Table S2). Correlation analyses showed systematically weaker associations between apoB and cardiometabolic risk factors in men (|r| = 0.001–0.17) than in women (|r| = 0.04–0.33). Among women, there was the strongest positive correlation between age and apoB (*r* = 0.33, *P* < 0.0001), but there was no significant correlation in men (*r* = -0.001, *P* = 0.51).

**Table 1:** Baseline characteristics across sex-specific quartiles (Q1-Q4) of apolipoprotein B (apoB)

	Whole population	Q1	Q2	Q3	Q4	*P* for trend ^a^
Variables	26803	6822	6684	6660	6637	
ApoB in men (mg/dL)	109 (22–325)	83 (22–93)	102 (94–109)	117 (110–126)	139 (127–325)	-
ApoB in women (mg/dL)	102 (29–309)	76 (29–86)	95 (87–102)	111 (103–120)	135 (121–309)	-
Age (years)	58.1 ± 7.63	55.9 ± 7.71	57.7 ± 7.59	59.0 ± 7.46	59.9 ± 7.15	< 0.0001
Sex (men, %)	10,453 (39.0)	2646 (38.8)	2620 (39.2)	2613 (39.2)	2574 (38.8)	0.9875
Body mass index (kg/m²)	25.7 ± 3.96	24.6 ± 3.68	25.4 ± 3.90	26.1 ± 3.94	26.8 ± 3.98	< 0.0001
Systolic blood pressure (mmHg)	141.1 ± 20.0	136.4 ± 19.6	139.8 ± 19.7	142.8 ± 19.7	145.6 ± 19.9	< 0.0001
Total leukocyte count (10^9^/L) ^b^	6.10 (5.20–7.30)	5.90 (5.10-7.00)	6.10 (5.20–7.20)	6.10 (5.20–7.40)	6.40 (5.40–7.50)	< 0.0001
Diabetes (%)	1163 (4.34)	226 (3.31)	264 (3.95)	293 (4.40)	380 (5.73)	< 0.0001
Anti-hypertensive medication (%)	4682 (17.5)	856 (12.6)	1061 (15.9)	1257 (18.9)	1508 (22.7)	< 0.0001
Lipid-lowering medication (%)	820 (3.06)	156 (2.29)	202 (3.02)	210 (3.15)	252 (3.80)	< 0.0001
Smokers (%)	7560 (28.2)	1678 (24.6)	1826 (27.3)	1947 (29.2)	2109 (31.8)	< 0.0001
High alcohol consumption (%)	1153 (4.30)	343 (5.03)	268 (4.01)	261 (3.92)	281 (4.23)	0.0239
High education (%)	8603 (32.1)	2742 (40.2)	2229 (33.4)	1962 (29.5)	1670 (25.2)	< 0.0001
Physical activity Quartiles 1	6643 (24.8)	1518 (22.3)	1635 (24.5)	1720 (25.8)	1770 (26.7)	< 0.0001
Physical activity Quartiles 2	6727 (25.1)	1714 (25.1)	1701 (25.5)	1661 (24.9)	1651 (24.9)	
Physical activity Quartiles 3	6713 (25.1)	1803 (26.4)	1636 (24.5)	1660 (24.9)	1614 (24.3)	
Physical activity Quartiles 4	6720 (25.1)	1787 (26.2)	1712 (25.6)	1619 (24.3)	1602 (24.1)	

Continuous variables are expressed as mean ± standard deviation; categorical variables are presented as n (%). Unless otherwise specified

^a^ Analyzed using linear regression or logistic regression

^b^ Total leukocyte is expressed as median (interquartile range) because of skewed distributions; *P* value was calculated using log-transformed values

### Risk of AF associated with apoB levels in men and women

**Fig. 2:**
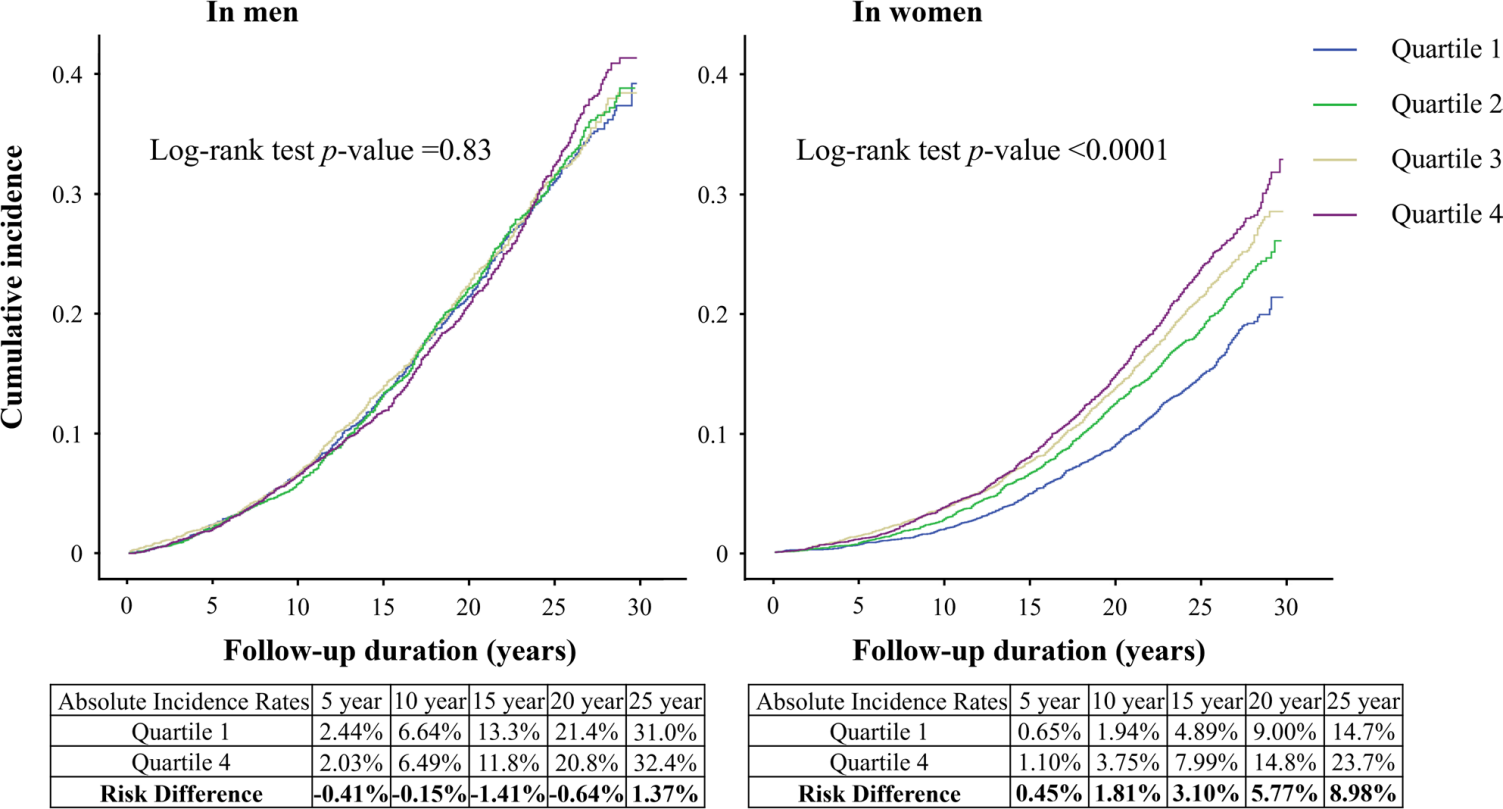
Unadjusted Sex-specific cumulative incidence curves by apolipoprotein B quartiles. AF, Atrial Fibrillation

During follow-up, 2,768 and 2,968 incident cases of AF were recorded in men and women, respectively. The median follow-up time for the entire cohort was 24.4 years (interquartile range [IQR]: 16.0–26.6), 21.2 years in men (IQR: 13.1–26.2) and 24.8 years in women (IQR: 18.3–26.7). A significant sex between apoB interaction was found for AF risk (*P* for interaction = 0.0038; HR for the interaction term = 0.92 per 1 SD increase in apoB, 95% confidence interval [CI] 0.87–0.97), suggesting a more pronounced inverse association in women than men. Kaplan-Meier plots stratified according to sex are shown in Fig. 2. In men, Cox regression models with no to full adjustment revealed no significant associations between apoB quartiles and AF. In contrast, in women, the unadjusted model (Model 1) showed a significant positive association between apoB quartiles and AF (highest versus lowest quartile: HR 1.65, 95% CI 1.49–1.84; *P* for trend < 0.0001). After adjustment for age (Model 2), this association was no longer significant (HR 0.93, 95% CI 0.83–1.03; *P* for trend = 0.09). Partial (Model 3) and full adjustment (Model 4) showed a significant inverse association (fully adjusted HR 0.77, 95% CI 0.69–0.86; *P* for trend < 0.0001), which remained after further adjustment for age at menopause (Model 5; Table 2). In women, the E-value for the observed fully adjusted HR (0.77, 95% CI 0.69–0.86) was 1.92 (with 1.59 for the lower confidence limit), meaning that an unmeasured confounder would have to be strongly associated (≥ 1.92-fold) with both apoB and AF to completely explain the observed association. In men, the E-value was lower at 1.43 with the lower confidence limit at 1.00, indicating a weaker buffering against unmeasured confounding. When apoB was considered as a continuous variable, a crude positive association in women became inverse after adjustment for age (per SD HR 0.96, 95% CI 0.92–1.00; *P* = 0.03) and became stronger with full covariate adjustment (HR 0.89, 95% CI 0.85–0.93; *P* < 0.0001). No violations of the proportional hazards assumption were found (global test *P* = 0.63), and Kaplan-Meier curves among women were nearly parallel. No multicollinearity was detected (all VIFs < 2), and results from the simplified model (Model 3) were similar to the full model (Model 4). No significant interactions were found between apoB and other covariates for either sex (all *P* for interaction > 0.05). RCS analyses showed a linear inverse relationship between apoB and AF in women (*P* for effect < 0.0001; *P* for nonlinearity = 0.48). In men, a borderline non-linear association was found (*P* for effect = 0.04; *P* for nonlinearity = 0.05; Figure S1), with *post-hoc* analyses indicating an inverse association at lower apoB concentrations (≤ 100 mg/dL: per SD HR 0.90, 95% CI 0.85–0.96; *P* = 0.0012), but not at higher concentrations (> 100 mg/dL: per SD HR 0.99, 95% CI 0.93–1.05; *P* = 0.68). The inverse apoB-AF association in women was robust in several sensitivity analyses, including using age as the time scale, modeling age using RCS, and age stratification (Table S3). In these analyses, no significant associations were found in men. Competing risk analyses taking into account non-AF mortality did not alter the results substantially (data not shown), and cumulative incidence function curves stratified by apoB quartiles showed clear sex-specific patterns (Figure S2). In the women, the highest apoB quartile was associated with the highest cumulative incidence of both death and AF, and death as a competing event had little effect on AF incidence. In men, the cumulative incidence of AF was similar in each apoB quartile. Exclusion of participants with baseline stroke or coronary events and treating incident events prior to AF as competing risks had a slightly greater strengthening impact on the inverse apoB-AF association in women (highest versus lowest quartile: HR 0.69, 95% CI 0.61–0.79; *P* for trend < 0.0001), but no significant link was found in men. Hardly any change was observed in HR values in sensitivity analyses that accounted for a history of thyroid-related diseases (data not shown). Consistent results were also obtained when excluding early AF events (Table S4) or restriction of follow-up (Table S5). Adjustment for CRP in a sub-cohort (*n* = 4,884) did not materially alter results. Pearson correlations between apoB and lipid parameters in a random subset (*n* = 5,003) were 0.76 for LDL, -0.31 for HDL, 0.68 for total cholesterol, and 0.43 for triglycerides (all *P* < 0.0001). Discordance analysis based on sex-specific medians of apoB and LDL in women showed HRs (95% CI) of 1.02 (0.77–1.35) for high-apoB/low-LDL, 0.66 (0.47–0.94) for low-apoB/high-LDL, and 0.73 (0.60–0.89) for high-apoB/high-LDL (*P* for trend = 0.0005), compared with the low-apoB/low-LDL. No associations were seen in men. Finally, sensitivity analyses with multiple imputation for missing data supported the robustness of the findings. The pooled HR per SD increase of apoB was 0.97 (95% CI 0.93–1.01; *P* = 0.11) in men and 0.89 (95% CI 0.86–0.93; *P* < 0.0001) in women.

**Table 2:** Incidence of atrial fibrillation in relation to apolipoprotein B (*n* = 26803)

	Quartiles of apolipoprotein B				*P* for trend ^a^	Per 1 SD ^b^	*P* ^a^
	Q1	Q2	Q3	Q4			
Men	2646	2620	2613	2574	-	10453	-
AF cases, n	692	682	694	700	-	2768	-
Incidence (per 1000 person-years)	13.5	13.6	13.8	14.1	-	13.7	-
Model 1	Reference	0.99 (0.89, 1.10)	1.02 (0.92, 1.14)	1.04 (0.94, 1.15)	0.7948	1.02 (0.98, 1.06)	0.3931
Model 2	Reference	0.96 (0.86, 1.07)	1.00 (0.90, 1.11)	1.04 (0.93, 1.15)	0.3655	1.02 (0.98, 1.06)	0.2556
Model 3	Reference	0.96 (0.86, 1.07)	0.99 (0.89, 1.11)	1.03 (0.92, 1.14)	0.4983	1.02 (0.98, 1.06)	0.3764
Model 4	Reference	0.91 (0.81, 1.01)	0.92 (0.82, 1.02)	0.91 (0.82, 1.01)	0.1041	0.97 (0.94, 1.01)	0.1513
Women	4176	4064	4047	4063	-	16350	-
AF cases, n	613	727	792	836	-	2968	-
Incidence (per 1000 person-years)	6.38	8.03	9.08	9.84	-	8.27	-
Model 1	Reference	1.27 (1.13, 1.41)	1.51 (1.36, 1.68)	1.65 (1.49, 1.84)	< 0.0001	1.17 (1.13, 1.21)	< 0.0001
Model 2	Reference	0.98 (0.88, 1.09)	0.96 (0.86, 1.07)	0.93 (0.83, 1.03)	0.0913	0.96 (0.92, 1.00)	0.0256
Model 3	Reference	0.97 (0.87, 1.09)	0.94 (0.84, 1.05)	0.90 (0.81, 1.01)	0.0326	0.95 (0.91, 0.99)	0.0076
Model 4	Reference	0.93 (0.83, 1.03)	0.85 (0.76, 0.95)	0.77 (0.69, 0.86)	< 0.0001	0.89 (0.86, 0.93)	< 0.0001
Model 5	Reference	0.92 (0.83, 1.03)	0.85 (0.76, 0.95)	0.77 (0.69, 0.86)	< 0.0001	0.89 (0.85, 0.93)	< 0.0001

Model 1: Crude model

Model 2: Adjusted for age

Model 3: Additionally adjusted smoking and drinking, education, and physical activity

Model 4: Additionally adjusted for body mass index, systolic blood pressure, total leukocyte count, diabetes, anti-hypertensive medication, and lipid-lowering medication

Model 5: Additionally adjusted for age at menopause in women

^a^ Analysis by Cox proportional hazards model. SD, standard deviation

^b^ The SD of apolipoprotein B levels is 25.4 mg/dL for men and 26.3 mg/dL for women

## Discussion

In this large, population-based study, elevated apoB levels were inversely related to the risk of AF in women, with this association remained largely consistent across multiple sensitivity analyses. In men, the association was of borderline non-linear type, with inverse associations mainly at lower apoB concentrations.

To the best of current knowledge, this is the first study to carry out detailed sex-specific longitudinal analyses of the association between apoB and AF. The inverse association in women is similar to a prospective study of 23,738 middle-aged and elderly women [10]. Notably, both studies showed that apoB was positively related to AF in unadjusted analyses, but this association was reversed after multivariable adjustment. Age likely served as a predominant confounder during this process. Advancing age is one of the most important risk factors for AF [17], and its known correlation with apoB levels in women [23], which was also noted in this study, highlights the importance of strict age adjustment. The large change in the crude association after adjustment for age underscores the confounding effect. Importantly, the different age-adjustment strategies used in the present study all support the conclusion that the inverse association in women is independent of age. In contrast, the null associations in men are consistent with a prospective study of 2,533 men [11]. Similarly, the Swedish Apolipoprotein-Related Mortality Risk (AMORIS) cohort study (*n* = 65,136) found no significant apoB- AF associations [12]. However, the AMORIS analysis did not include sex stratification and controlled for a small number of covariates (age, sex and socioeconomic status). Of note, the lack of significant associations in unadjusted and age-adjusted models in this study is consistent with the AMORIS findings.

Previous studies on the role of apoB in AF are limited. One possible mechanism that explains the observed inverse association between apoB, the core structural apolipoprotein of LDL, and AF could be linked to the stabilization effect of cholesterol on myocardial membranes. This effect may modulate the function of ion channels and stabilize membrane potential [7, 24], which may affect the susceptibility to AF. Supporting this hypothesis, metabolic conditions have been associated with atrial electrical remodeling [25]. The discordance analysis further supports this idea by demonstrating a greater inverse association in women with low apoB but high LDL than those with high apoB and low LDL, implying a cholesterol-dependent membrane stabilization effect rather than a pure particle number effect. These findings extend the previously proposed “cholesterol paradox” in AF [6, 7] to apoB. Although direct evidence for sex-specific effects of apoB in AF are lacking, other lipids, including total cholesterol [26] and HDL [27] have shown sex-specific associations with AF risk. Documented sex differences in atrial electrophysiology, including calcium handling and ion channel regulation [28], may be the basis for these disparities. Hormonal factors, especially estrogen, also affect sex-specific lipid metabolism and adipose distribution [7, 29]. In addition, anthropometric measures are also strongly related to AF risk [30, 31], with distinct sex-specific differences in the effect [31, 32]. Collectively, these factors might be responsible for the observed sex-specific association between lipids and AF. Although age at menopause is associated with the risk of AF in women [33], adjustment for this variable had little effect on the association, suggesting that confounding is limited. In men, the inverse apoB-AF association was seen only in the lower apoB category. Whether this is due to residual confounding (e.g.., poorer nutritional status) or attenuation due to more pronounced metabolic disturbances at higher apoB concentrations is unclear. Overall, these mechanistic explanations are speculative and need to be investigated further.

The strong model dependence of apoB-AF associations in women raises questions about the robustness of the findings and suggests that residual confounding or unmeasured factors may contribute. Given the observational nature of the data, the inverse association might be affected by conditions such as hyperthyroidism or systemic inflammation, which are both associated with higher risk of AF [21, 34] and lower LDL levels [22, 35]. Nevertheless, in this study, multivariable adjustments, extensive sensitivity analysis, and E-value calculations all support the relative robustness of the inverse association in women against potential residual confounding. In contrast, the null association in men was more prone to unmeasured confounding, adding to the support for sex-specific differences in apoB-AF associations found in this study.

Previous Mendelian randomization (MR) studies on apoB and AF have shown either non-significant [36–38] or positive associations [39]. However, MR studies are reflective of lifelong exposure to genetically determined apoB levels, which may be different in observational studies that measure apoB at discrete time points and are influenced by lifestyle factors, medical treatments and age-related changes. Additionally, MR analyses tend not to stratify by sex, nor do they control for time-varying confounders, for example, age. In contrast, the present study used covariate-adjusted analyses, which included these complexities of real-world settings. Furthermore, pathophysiological heterogeneity in AF, such as differences in coronary and non-coronary etiologies, may contribute to the discrepancy between MR and observational findings. Collectively, these results provide unique insights into sex-specific apoB-AF associations in real-world conditions.

### Strengths and limitations

This study has a number of strengths, such as a large sample size, long-term prospective follow-up, and validated outcome assessments. The findings add to the understanding of sex-specific apoB-AF associations in real-world settings and complement previous observational and genetic studies, which tended to lack comprehensive covariate adjustment or sex-stratified analyses. Several limitations are to be considered. First, residual confounding due to unmeasured factors, such as sex-specific hormonal influences, thyroid hormones, genetic variants, detailed inflammatory biomarkers, or Lipoprotein (a), a cardiovascular risk factor with thrombogenic and pro-inflammatory effects, cannot be completely ruled out. Other lipid parameters were not included in adjustment models as they are biologically correlated with apoB, and adjusting for them could partition shared variance and distort true effect estimates. Second, analyses were based on one baseline measurement of apoB and covariates, which limited the ability to account for intra-individual variability over time or for changes in factors such as lipid-lowering therapy over follow-up. ApoB was measured in serum samples stored at -80 °C for more than 10 years and although their long-term stability has not been independently confirmed, previous studies have supported the stability of lipid biomarkers, including apoB, in frozen serum [40, 41]. The predictive value of these biobank measurements has been shown in several previous studies [42–44], which supports their reliability. Third, AF classification did not permit differentiation of subtypes (e.g., paroxysmal vs. persistent), which may have different pathophysiological mechanisms. Fourth, the study population was largely of Western European ethnicity and participants were likely to represent a generally healthier segment of the population, limiting generalizability to other ethnic groups or clinical contexts. Fifth, although the exclusion of participants with pre-existing AF is standard practice, undiagnosed asymptomatic cases may have introduced survival bias. Additionally, changing treatment practices during follow-up may also have introduced variability, though this reflects real-world conditions. Finally, because of the observational design, causal inferences cannot be made. While multiple testing in subgroup and sex-stratified analyses could theoretically increase type I error, the consistency of results among subgroups and highly significant *P*-values seen in women (< 0.0001) are consistent with the robustness of results. The exploratory nature of sensitivity analyses is also recognized, and these results are therefore suggested to be interpreted as hypothesis-generating rather than definitive. Overall, these findings represent associations, not causation, and additional studies in diverse cohorts with serial biomarker measurements are warranted to validate these associations and provide insight into underlying mechanisms.

## Conclusion

In this population-based prospective cohort study, sex-specific associations were found between apoB levels and AF incidence with a linear inverse association for women and a borderline non-linear association for men with inverse associations mainly at lower apoB concentrations. These results indicate that clinicians may need to take sex-specific apoB levels into account when assessing AF risk, especially in women. Although the study reports associations and relative risks, the causal nature of these relationships is still uncertain and should be interpreted with caution. Future studies are needed to confirm these sex-specific trends in independent cohorts, to understand the underlying biological mechanisms, and to examine whether lipid-modifying interventions can affect AF risk in high-risk populations.

## Supplementary Information

Supplementary Material 1.

## Data Availability

Data are available upon reasonable request and with permission of Malmö Diet and Cancer steering committee. Details can be found on the Lund University website. (https://www.malmo-kohorter.lu.se/malmo-cohorts).

## Abbreviations

AF: Atrial fibrillation
AMORIS: Swedish Apolipoprotein-Related Mortality Risk
apoB: Apolipoprotein B
CI: Confidence interval
CRP: C-reactive protein
HDL: High-density lipoprotein
HR: Hazard ratio
ICD: International Classification of Diseases
LDL: Low-density lipoprotein
MDCS: Malmö Diet and Cancer Study
MR: Mendelian randomization
RCS: Restricted cubic spline
SD: Standard deviation
VIF: Variance inflation factor

## References

[1] Joglar JA, Chung MK, Armbruster AL, Benjamin EJ, Chyou JY, Cronin EM, et al. 2023 ACC/AHA/ACCP/HRS Guideline for the Diagnosis and Management of Atrial Fibrillation: A Report of the American College of Cardiology/American Heart Association Joint Committee on Clinical Practice Guidelines. Circulation. 2024;149:e1–156. 38033089 10.1161/CIR.0000000000001193 PMC11095842

[2] Ference BA, Ginsberg HN, Graham I, Ray KK, Packard CJ, Bruckert E, et al. Low-density lipoproteins cause atherosclerotic cardiovascular disease. 1. Evidence from genetic, epidemiologic, and clinical studies. A consensus statement from the European Atherosclerosis Society Consensus Panel. Eur Heart J. 2017;38:2459–72. 28444290 10.1093/eurheartj/ehx144 PMC5837225

[3] Van Gelder IC, Rienstra M, Bunting KV, Casado-Arroyo R, Caso V, Crijns H, et al. 2024 ESC Guidelines for the management of atrial fibrillation developed in collaboration with the European Association for Cardio-Thoracic Surgery (EACTS). Eur Heart J. 2024;45:3314–414. 39210723 10.1093/eurheartj/ehae176

[4] Boren J, Packard CJ, Binder CJ. Apolipoprotein B-containing lipoproteins in atherogenesis. Nat Rev Cardiol. 2025;22:399–413. 39743565 10.1038/s41569-024-01111-0

[5] Borén J, Chapman MJ, Krauss RM, Packard CJ, Bentzon JF, Binder CJ, et al. Low-density lipoproteins cause atherosclerotic cardiovascular disease: pathophysiological, genetic, and therapeutic insights: a consensus statement from the European Atherosclerosis Society Consensus Panel. Eur Heart J. 2020;41:2313–30. 32052833 10.1093/eurheartj/ehz962 PMC7308544

[6] Suzuki S. Cholesterol paradox in atrial fibrillation. Circ J. 2011;75:2749–50. 22027365 10.1253/circj.cj-11-1134

[7] Ding WY, Protty MB, Davies IG, Lip GYH. Relationship between lipoproteins, thrombosis, and atrial fibrillation. Cardiovasc Res. 2022;118:716–31. 33483737 10.1093/cvr/cvab017 PMC8859639

[8] Zhong X, Jiao H, Zhao D, Teng J. Association between serum apolipoprotein B and atrial fibrillation: a case-control study. Sci Rep. 2022;12:9597. 35688870 10.1038/s41598-022-13773-2 PMC9187736

[9] Zhong X, Jiao H, Zhao D, Teng J, Yang M. Case-Control Study to Investigate the Association Between Serum Apolipoprotein B/A1 Ratio and Atrial Fibrillation by Sex in 920 Patients from China. Med Sci Monit. 2022;28:e936425. 35567295 10.12659/MSM.936425 PMC9116146

[10] Mora S, Akinkuolie AO, Sandhu RK, Conen D, Albert CM. Paradoxical association of lipoprotein measures with incident atrial fibrillation. Circ Arrhythm Electrophysiol. 2014;7:612–9. 24860180 10.1161/CIRCEP.113.001378 PMC4591535

[11] Tajik B, Tuomainen TP, Jarroch R, Kauhanen J, Lip GYH, Isanejad M. Lipid levels, apolipoproteins, and risk of incident atrial fibrillation in men: A report from the Kuopio Ischaemic Heart Disease Risk Factor Study (KIHD). J Clin Lipidol. 2022;16:447–54. 35525793 10.1016/j.jacl.2022.04.003

[12] Ding M, Wennberg A, Gigante B, Walldius G, Hammar N, Modig K. Lipid levels in midlife and risk of atrial fibrillation over 3 decades-Experience from the Swedish AMORIS cohort: A cohort study. PLoS Med. 2022;19:e1004044. 35951514 10.1371/journal.pmed.1004044 PMC9371362

[13] Berglund G, Elmstahl S, Janzon L, Larsson SA. The Malmo Diet and Cancer Study. Design and feasibility. J Intern Med. 1993;233:45–51. 8429286 10.1111/j.1365-2796.1993.tb00647.x

[14] Pero RW, Olsson A, Berglund G, Janzon L, Larsson SA, Elmståhl S. The Malmö biological bank. J Intern Med. 1993;233:63–7. 8429289 10.1111/j.1365-2796.1993.tb00650.x

[15] Smith JG, Platonov PG, Hedblad B, Engstrom G, Melander O. Atrial fibrillation in the Malmo Diet and Cancer study: a study of occurrence, risk factors and diagnostic validity. Eur J Epidemiol. 2010;25:95–102. 19936945 10.1007/s10654-009-9404-1

[16] Tzeis S, Gerstenfeld EP, Kalman J, Saad EB, Shamloo AS, Andrade JG, et al. 2024 European Heart Rhythm Association/Heart Rhythm Society/Asia Pacific Heart Rhythm Society/Latin American Heart Rhythm Society expert consensus statement on catheter and surgical ablation of atrial fibrillation. Heart Rhythm. 2024;21:e31–149. 38597857 10.1016/j.hrthm.2024.03.017

[17] Staerk L, Sherer JA, Ko D, Benjamin EJ, Helm RH. Atrial Fibrillation: Epidemiology, Pathophysiology, and Clinical Outcomes. Circ Res. 2017;120:1501–17. 28450367 10.1161/CIRCRESAHA.117.309732 PMC5500874

[18] Lunn M, McNeil D. Applying Cox regression to competing risks. Biometrics. 1995;51:524–32. 7662841

[19] Bao X, Borne Y, Johnson L, Muhammad IF, Persson M, Niu K, et al. Comparing the inflammatory profiles for incidence of diabetes mellitus and cardiovascular diseases: a prospective study exploring the ‘common soil’ hypothesis. Cardiovasc Diabetol. 2018;17:87. 29895294 10.1186/s12933-018-0733-9 PMC5996509

[20] Frederiksen TC, Dahm CC, Preis SR, Lin H, Trinquart L, Benjamin EJ, et al. The bidirectional association between atrial fibrillation and myocardial infarction. Nat Rev Cardiol. 2023;20:631–44. 37069297 10.1038/s41569-023-00857-3 PMC11380523

[21] Wiersinga WM, Poppe KG, Effraimidis G. Hyperthyroidism: aetiology, pathogenesis, diagnosis, management, complications, and prognosis. Lancet Diabetes Endocrinol. 2023;11:282–98. 36848916 10.1016/S2213-8587(23)00005-0

[22] Duntas LH, Brenta G. The effect of thyroid disorders on lipid levels and metabolism. Med Clin North Am. 2012;96:269–81. 22443975 10.1016/j.mcna.2012.01.012

[23] Wu B, Fan B, Qu Y, Li C, Chen J, Liu Y, et al. Trajectories of Blood Lipids Profile in Midlife Women: Does Menopause Matter? J Am Heart Assoc. 2023;12:e030388. 37947109 10.1161/JAHA.123.030388 PMC10727300

[24] Dart C. Lipid microdomains and the regulation of ion channel function. J Physiol. 2010;588:3169–78. 20519314 10.1113/jphysiol.2010.191585 PMC2976012

[25] Ates MS, Sokmen E. Electrocardiographic P-Wave Indices in Metabolic Dysfunction-Associated Fatty Liver Disease and Their Relationship to Hepatic Fibrosis Risk. J Clin Med. 2025;14:4650. 40649039 10.3390/jcm14134650 PMC12250510

[26] Magnussen C, Niiranen TJ, Ojeda FM, Gianfagna F, Blankenberg S, Njølstad I, et al. Sex Differences and Similarities in Atrial Fibrillation Epidemiology, Risk Factors, and Mortality in Community Cohorts: Results From the BiomarCaRE Consortium (Biomarker for Cardiovascular Risk Assessment in Europe). Circulation. 2017;136:1588–97. 29038167 10.1161/CIRCULATIONAHA.117.028981 PMC5657474

[27] Watanabe H, Tanabe N, Yagihara N, Watanabe T, Aizawa Y, Kodama M. Association between lipid profile and risk of atrial fibrillation. Circ J. 2011;75:2767–74. 21914959 10.1253/circj.cj-11-0780

[28] Smith CER, Ni H, Grandi E. Sex Differences in Electrophysiology and Calcium Handling in Atrial Health and Fibrillation. Annu Rev Physiol. 2025;87:1–24. 39441881 10.1146/annurev-physiol-022724-104938 PMC12498256

[29] Palmisano BT, Zhu L, Eckel RH, Stafford JM. Sex differences in lipid and lipoprotein metabolism. Mol Metab. 2018;15:45–55. 29858147 10.1016/j.molmet.2018.05.008 PMC6066747

[30] Shojaei S, Radkhah H, Akhlaghipour I, Shad AN, Azarboo A, Mousavi A. Waist circumference and body surface area and the risk of developing new-onset atrial fibrillation: A systematic review and meta-analysis of observational studies. Heart Lung. 2025;72:1–12. 40088585 10.1016/j.hrtlng.2025.02.008

[31] Lu Z, Geurts S, Arshi B, Tilly MJ, Aribas E, Roeters van Lennep J, et al. Longitudinal Anthropometric Measures and Risk of New-Onset Atrial Fibrillation Among Community-Dwelling Men and Women. Mayo Clin Proc. 2022;97:1501–11. 35691705 10.1016/j.mayocp.2021.12.018

[32] Siddiqi HK, Vinayagamoorthy M, Gencer B, Ng C, Pester J, Cook NR, et al. Sex Differences in Atrial Fibrillation Risk: The VITAL Rhythm Study. JAMA Cardiol. 2022;7:1027–35. 36044209 10.1001/jamacardio.2022.2825 PMC9434484

[33] Shin J, Han K, Jung JH, Park HJ, Kim W, Huh Y, et al. Age at menopause and risk of heart failure and atrial fibrillation: a nationwide cohort study. Eur Heart J. 2022;43:4148–57. 36239217 10.1093/eurheartj/ehac364

[34] Hu YF, Chen YJ, Lin YJ, Chen SA. Inflammation and the pathogenesis of atrial fibrillation. Nat Rev Cardiol. 2015;12:230–43. 25622848 10.1038/nrcardio.2015.2

[35] Herbert KE, Erridge C. Regulation of low-density lipoprotein cholesterol by intestinal inflammation and the acute phase response. Cardiovasc Res. 2018;114:226–32. 29206916 10.1093/cvr/cvx237

[36] Jiang Q, Qin D, Yang L, Lin Y, Zhai L, Zhang Y, et al. Causal effects of plasma lipids on the risk of atrial fibrillation: A multivariable mendelian randomization study. Nutr Metab Cardiovasc Dis. 2021;31:1569–78. 33814236 10.1016/j.numecd.2021.02.011

[37] Schmidt AF, Joshi R, Gordillo-Marañón M, Drenos F, Charoen P, Giambartolomei C, et al. Biomedical consequences of elevated cholesterol-containing lipoproteins and apolipoproteins on cardiovascular and non-cardiovascular outcomes. Commun Med (Lond). 2023;3:9. 36670186 10.1038/s43856-022-00234-0 PMC9859819

[38] Yang S, Pudasaini R, Zhi H, Wang L. The Relationship between Blood Lipids and Risk of Atrial Fibrillation: Univariable and Multivariable Mendelian Randomization Analysis. Nutrients. 2021;14:181. 35011056 10.3390/nu14010181 PMC8746968

[39] Zhang Z, Li L, Zhang Z, Hu Z, Xiong Y, Zhou L, et al. Associations of 50 modifiable risk factors with atrial fibrillation using Mendelian randomization analysis. Eur J Clin Invest. 2024;54:e14194. 38438337 10.1111/eci.14194

[40] Jansen EHJM, Beekhof PK, Schenk E. Long Term Stability of Parameters of Lipid Metabolism in Frozen Human Serum: Triglycerides, Free Fatty Acids, Total-, HDL- and LDL-cholesterol, Apolipoprotein-A1 and B. J Mol Biomark Diagn. 2014;5:4.

[41] Muzakova V, Beekhof PK, Jansen E. Very long-term stability of lipid biomarkers in human serum. Anal Biochem. 2020;597:113695. 32201135 10.1016/j.ab.2020.113695

[42] Fritz J, Shiffman D, Melander O, Tada H, Ulmer H. Metabolic Mediators of the Effects of Family History and Genetic Risk Score on Coronary Heart Disease-Findings From the Malmo Diet and Cancer Study. J Am Heart Assoc. 2017;6:e005254. 28320750 10.1161/JAHA.116.005254 PMC5524031

[43] Landenhed M, Engstrom G, Gottsater A, Caulfield MP, Hedblad B, Newton-Cheh C, et al. Risk profiles for aortic dissection and ruptured or surgically treated aneurysms: a prospective cohort study. J Am Heart Assoc. 2015;4:e001513. 25609416 10.1161/JAHA.114.001513 PMC4330075

[44] Wadstrom K, Jacobsson LTH, Mohammad AJ, Warrington KJ, Matteson EL, Turesson C. Apolipoproteins and the risk of giant cell arteritis-a nested case-control study. Arthritis Res Ther. 2024;26:37. 38281009 10.1186/s13075-024-03273-1 PMC10821258

