# Supplementary material for: A population-based study of the sex-specific associations between apolipoprotein B and incidence of atrial fibrillation
Source: Lipids Health Dis. 2026 Feb 23;25:93. doi: 10.1186/s12944-026-02905-6 (PMC13037237; doi:10.1186/s12944-026-02905-6)
Supplement: Supplementary file 1 — Supplementary Material 1. [file 12944_2026_2905_MOESM1_ESM.docx]

**Supplementary materials**

**Table S1. Baseline characteristics between the excluded and included participants ^a^.**

**Table S2. Baseline characteristics between men and women.**

**Table S3. Sensitivity analyses of the apolipoprotein B and atrial fibrillation association using different adjustments for age (n=26803).**

**Table S4. Incidence of atrial fibrillation in relation to apolipoprotein B excluding participants with incident cases within the first 5 years of follow-up (n=26432).**

**Table S5. Incidence of atrial fibrillation in relation to apolipoprotein B with 15-year maximum follow-up (n=26803).**

**Figure S1. Restricted cubic spline analysis of sex-specific associations between apolipoprotein B and atrial fibrillation incidence.**

**Figure S2. Sex-specific cumulative incidence curves by apolipoprotein B quartiles.** AF, atrial fibrillation.

| **Table S1. Baseline characteristics between the excluded and included participants ^a^.** | | | | | |
| --- | --- | --- | --- | --- | --- |
|  |  | **Excluded participants** | **Included participants** | *P* ^b^ | SMD ^c^ |
| Variables | N | 1347 | 26803 |  |  |
| ApoB in men (mg/dL) | 11056 | 113.6 ± 27.2 | 110.6 ± 25.4 | 0.0413 | 0.1130 |
| ApoB in women (mg/dL) | 17094 | 110.5 ± 27.0 | 104.8 ± 26.3 | <0.0001 | 0.2140 |
| Age (years) | 28150 | 57.9 ± 6.60 | 58.1 ± 7.60 | 0.4300 | -0.0280 |
| Sex (men, %) | 28150 | 603 (44.8) | 10453 (39) | <0.0001 | - |
| Body mass index (kg/m²) | 28106 | 26.4 ± 4.30 | 25.7 ± 4.00 | <0.0001 | 0.1690 |
| Systolic blood pressure (mmHg) | 28108 | 141.7 ± 20.0 | 141.1 ± 20.0 | 0.2714 | 0.0300 |
| Total leukocyte count (10^9^/L) ^d^ | 28074 | 6.30 (5.30-7.50) | 6.10 (5.20-7.30) | 0.0620 | 0.0450 |
| Diabetes (%) | 28150 | 99 (7.35) | 1163 (4.34) | <0.0001 | - |
| Anti-hypertensive medication (%) | 28150 | 200 (14.9) | 4682 (17.5) | 0.0133 | - |
| Lipid-lowering medication (%) | 28150 | 28 (2.08) | 820 (3.06) | 0.0412 | - |
| Smokers (%) | 27831 | 333 (32.4) | 7560 (28.2) | 0.0035 | - |
| High alcohol consumption (%) | 27831 | 43 (4.18) | 1153 (4.3) | 0.8554 | - |
| High education (%) | 27774 | 244 (25.1) | 8603 (32.1) | <0.0001 | - |
| Physical activity Quartiles 1 | 27650 | 266 (31.4) | 6643 (24.8) | 0.0008 | - |
| Physical activity Quartiles 2 |  | 191 (22.6) | 6727 (25.1) |  |  |
| Physical activity Quartiles 3 |  | 194 (22.9) | 6713 (25.1) |  |  |
| Physical activity Quartiles 4 |  | 196 (23.1) | 6720 (25.1) |  |  |
| Continuous variables are expressed as mean ± standard deviation; categorical variables are presented as n (%). Unless otherwise specified. SMD, standardized mean difference. | | | | | |
| ^a^ Subjects with baseline atrial fibrillation (n=299) were excluded from this comparison. | | | | | |
| ^b^ Analyzed using linear regression or logistic regression. | | | | | |
| ^c^ Calculated using Hedges' g formulas. | | | | | |
| ^d^ Total leukocyte is expressed as median (interquartile range) because of skewed distributions; *P* value was calculated using log-transformed values. | | | | | |

| **Table S2. Baseline characteristics between men and women.** | | | | |
| --- | --- | --- | --- | --- |
|  | **Whole population** | **Men** | **Women** | *P* ^a^ |
| Variables | 26803 | 10453 | 16350 |  |
| ApoB (mg/dL, range) | 105 (22-325) | 109 (22-325) | 102 (29-309) | <0.0001 |
| Age (years) | 58.1 ± 7.63 | 59.2 ± 7.06 | 57.4 ± 7.89 | <0.0001 |
| Body mass index (kg/m²) | 25.7 ± 3.96 | 26.2 ± 3.44 | 25.4 ± 4.23 | <0.0001 |
| Systolic blood pressure (mmHg) | 141.1 ± 20.0 | 144 ± 19.33 | 139.2 ± 20.2 | <0.0001 |
| Total leukocyte count (10^9^/L) ^b^ | 6.10 (5.20-7.30) | 6.10 (5.20-7.20) | 6.10 (5.20-7.30) | 0.0653 |
| Diabetes (%) | 1163 (4.34) | 603 (5.77) | 560 (3.43) | <0.0001 |
| Anti-hypertensive medication (%) | 4682 (17.5) | 2085 (20) | 2597 (15.9) | <0.0001 |
| Lipid-lowering medication (%) | 820 (3.06) | 483 (4.62) | 337 (2.06) | <0.0001 |
| Smokers (%) | 7560 (28.2) | 2977 (28.5) | 4583 (28.0) | 0.4248 |
| High alcohol consumption (%) | 1153 (4.30) | 763 (7.30) | 390 (2.39) | <0.0001 |
| High education (%) | 8603 (32.1) | 3628 (34.7) | 4975 (30.4) | <0.0001 |
| Physical activity Quartiles 1 | 6643 (24.8) | 2635 (25.2) | 4008 (24.5) | 0.0362 |
| Physical activity Quartiles 2 | 6727 (25.1) | 2531 (24.2) | 4196 (25.7) |  |
| Physical activity Quartiles 3 | 6713 (25.1) | 2485 (23.8) | 4228 (25.9) |  |
| Physical activity Quartiles 4 | 6720 (25.1) | 2802 (26.8) | 3918 (24.0) |  |
| Continuous variables are expressed as mean ± standard deviation; categorical variables are presented as n (%). Unless otherwise specified. | | | | |
| ^a^ Analyzed using linear regression or logistic regression. | | | | |
| ^b^ Total leukocyte is expressed as median (interquartile range) because of skewed distributions; *P* value was calculated using log-transformed values. | | | | |

| **Table S3. Sensitivity analyses of the apolipoprotein B and atrial fibrillation association using different adjustments for age (n=26803).** | | | | | | | | |
| --- | --- | --- | --- | --- | --- | --- | --- | --- |
|  | **Quartiles of apolipoprotein B** | | | | *P* for trend ^a^ |  | Per 1 SD | *P* ^a^ |
|  | Q1 | Q2 | Q3 | Q4 |  |  |  |  |
| **Men** |  |  |  |  |  |  |  |  |
| **Age as covariate (primary analysis)** | Reference | 0.91 (0.81, 1.01) | 0.92 (0.82, 1.02) | 0.91 (0.82, 1.01) | 0.1041 |  | 0.97 (0.94, 1.01) | 0.1513 |
| **Age as time-scale** | Reference | 0.90 (0.81, 1.01) | 0.92 (0.83, 1.02) | 0.92 (0.83, 1.02) | 0.1600 |  | 0.98 (0.94, 1.02) | 0.2310 |
| **Age as time-dependent covariable** | Reference | 0.91 (0.82, 1.01) | 0.92 (0.83, 1.02) | 0.91 (0.82, 1.02) | 0.1259 |  | 0.97 (0.94, 1.01) | 0.1750 |
| **Age-stratified analysis** |  |  |  |  |  |  |  |  |
| age <60 y (n=5398) | Reference | 0.90 (0.76, 1.07) | 0.94 (0.79, 1.11) | 0.95 (0.81, 1.12) | 0.6929 |  | 0.98 (0.93, 1.04) | 0.5840 |
| age ≥60 y (n=5055) | Reference | 0.91 (0.79, 1.04) | 0.90 (0.78, 1.04) | 0.87 (0.76, 1.01) | 0.0692 |  | 0.96 (0.91, 1.01) | 0.1497 |
|  |  |  |  |  |  |  |  |  |
| **Women** |  |  |  |  |  |  |  |  |
| **Age as covariate (primary analysis)** | Reference | 0.92 (0.83, 1.03) | 0.85 (0.76, 0.95) | 0.77 (0.69, 0.86) | <0.0001 |  | 0.89 (0.85, 0.93) | <0.0001 |
| **Age as time-scale** | Reference | 0.91 (0.81, 1.01) | 0.83 (0.74, 0.92) | 0.74 (0.67, 0.83) | <0.0001 |  | 0.88 (0.85, 0.92) | <0.0001 |
| **Age as time-dependent covariable** | Reference | 0.93 (0.83, 1.04) | 0.86 (0.77, 0.96) | 0.78 (0.69, 0.87) | <0.0001 |  | 0.89 (0.86, 0.93) | <0.0001 |
| **Age-stratified analysis** |  |  |  |  |  |  |  |  |
| age <60 y (n=9716) | Reference | 0.90 (0.77, 1.05) | 0.91 (0.77, 1.07) | 0.69 (0.58, 0.83) | 0.0005 |  | 0.86 (0.81, 0.92) | <0.0001 |
| age ≥60 y (n=6634) | Reference | 0.93 (0.79, 1.09) | 0.80 (0.69, 0.94) | 0.78 (0.67, 0.91) | <0.0001 |  | 0.90 (0.86, 0.95) | <0.0001 |
| ^a^ Analysis by Cox proportional hazards model, with multivariable adjustment. SD, standard deviation. | | | | | | | | |

| **Table S4. Incidence of atrial fibrillation in relation to apolipoprotein B excluding participants with incident cases within the first 5 years of follow-up (n=26432).** | | | | | | | | |
| --- | --- | --- | --- | --- | --- | --- | --- | --- |
|  | **Quartiles of apolipoprotein B** | | | | *P* for trend ^a^ |  | Per 1 SD | *P* ^a^ |
|  | Q1 | Q2 | Q3 | Q4 |  |  |  |  |
| **Men** | 2585 | 2567 | 2553 | 2526 | - |  | 10231 | - |
| AF cases, n | 631 | 629 | 634 | 652 | - |  | 2546 | - |
| Incidence (per 1000 person-years) | 12.3 | 12.6 | 12.6 | 13.2 | - |  | 12.7 | - |
| Model 1 | Reference | 1.00 (0.89, 1.11) | 1.02 (0.92, 1.14) | 1.06 (0.95, 1.18) | 0.2449 |  | 1.03 (0.99, 1.07) | 0.1431 |
| Model 2 | Reference | 0.97 (0.86, 1.08) | 1.00 (0.89, 1.11) | 1.05 (0.94, 1.18) | 0.2714 |  | 1.03 (0.99, 1.08) | 0.0947 |
| Model 3 | Reference | 0.91 (0.82, 1.02) | 0.92 (0.82, 1.02) | 0.93 (0.83, 1.03) | 0.2147 |  | 0.99 (0.95, 1.03) | 0.4818 |
|  |  |  |  |  |  |  |  |  |
| **Women** | 4151 | 4037 | 3993 | 4020 | - |  | 16201 | - |
| AF cases, n | 588 | 700 | 738 | 793 | - |  | 2819 | - |
| Incidence (per 1000 person-years) | 6.12 | 7.74 | 8.48 | 9.35 | - |  | 7.87 | - |
| Model 1 | Reference | 1.27 (1.14, 1.43) | 1.48 (1.32, 1.65) | 1.65 (1.48, 1.83) | <0.0001 |  | 1.17 (1.13, 1.21) | <0.0001 |
| Model 2 | Reference | 0.99 (0.89, 1.11) | 0.95 (0.84, 1.06) | 0.93 (0.83, 1.04) | 0.0939 |  | 0.96 (0.92, 1.00) | 0.0452 |
| Model 3 | Reference | 0.94 (0.84, 1.05) | 0.84 (0.75, 0.94) | 0.77 (0.69, 0.87) | <0.0001 |  | 0.89 (0.86, 0.93) | <0.0001 |
| Model 4 | Reference | 0.94 (0.84, 1.05) | 0.84 (0.75, 0.94) | 0.77 (0.69, 0.87) | <0.0001 |  | 0.89 (0.86, 0.93) | <0.0001 |
| ^a^ Analysis by Cox proportional hazards model. SD, standard deviation. | | | | | | | | |
| Model 1: Crude model. | | | | | | | | |
| Model 2: Adjusted for age. | | | | | | | | |
| Model 3: Additionally adjusted for body mass index, systolic blood pressure, total leukocyte count, diabetes, anti-hypertensive medication, lipid-lowering medication, smoking, drinking, education, and physical activity. | | | | | | | | |
| Model 4: Additionally adjusted for age at menopause in women. | | | | | | | | |

| **Table S5. Incidence of atrial fibrillation in relation to apolipoprotein B with 15-year maximum follow-up (n=26803).** | | | | | | | | |
| --- | --- | --- | --- | --- | --- | --- | --- | --- |
|  | **Quartiles of apolipoprotein B** | | | | *P* for trend ^a^ |  | Per 1 SD | *P* ^a^ |
|  | Q1 | Q2 | Q3 | Q4 |  |  |  |  |
| **Men** | 2646 | 2620 | 2613 | 2574 | - |  | 10453 | - |
| AF cases, n | 314 | 310 | 326 | 272 | - |  | 1222 | - |
| Incidence (per 1000 person-years) | 9 | 9 | 9.5 | 8.1 | - |  | 8.9 | - |
| Model 1 | Reference | 0.97 (0.83, 1.14) | 1.07 (0.92, 1.25) | 0.90 (0.77, 1.06) | 0.3259 |  | 0.96 (0.90, 1.01) | 0.133 |
| Model 2 | Reference | 0.96 (0.82, 1.12) | 1.07 (0.92, 1.25) | 0.93 (0.79, 1.10) | 0.6195 |  | 0.97 (0.92, 1.03) | 0.3575 |
| Model 3 | Reference | 0.91 (0.77, 1.06) | 0.99 (0.85, 1.16) | 0.82 (0.70, 0.97) | 0.0355 |  | 0.93 (0.87, 0.98) | 0.01 |
|  |  |  |  |  |  |  |  |  |
| **Women** | 4176 | 4064 | 4047 | 4063 | - |  | 16350 | - |
| AF cases, n | 194 | 254 | 287 | 299 | - |  | 1034 | - |
| Incidence (per 1000 person-years) | 3.26 | 4.41 | 5.1 | 5.35 | - |  | 4.51 | - |
| Model 1 | Reference | 1.31 (1.08, 1.59) | 1.65 (1.37, 1.98) | 1.66 (1.39, 2.00) | <0.0001 |  | 1.17 (1.10, 1.24) | <0.0001 |
| Model 2 | Reference | 0.94 (0.77, 1.14) | 0.93 (0.77, 1.12) | 0.82 (0.68, 0.99) | 0.0344 |  | 0.93 (0.87, 0.99) | 0.0191 |
| Model 3 | Reference | 0.88 (0.73, 1.07) | 0.82 (0.68, 1.00) | 0.68 (0.56, 0.82) | <0.0001 |  | 0.86 (0.81, 0.92) | <0.0001 |
| Model 4 | Reference | 0.88 (0.73, 1.07) | 0.83 (0.68, 1.00) | 0.68 (0.56, 0.82) | <0.0001 |  | 0.86 (0.81, 0.92) | <0.0001 |
| ^a^ Analysis by Cox proportional hazards model. SD, standard deviation. | | | | | | | | |
| Model 1: Crude model. | | | | | | | | |
| Model 2: Adjusted for age. | | | | | | | | |
| Model 3: Additionally adjusted for body mass index, systolic blood pressure, total leukocyte count, diabetes, anti-hypertensive medication, lipid-lowering medication, smoking, drinking, education, and physical activity. | | | | | | | | |
| Model 4: Additionally adjusted for age at menopause in women. | | | | | | | | |


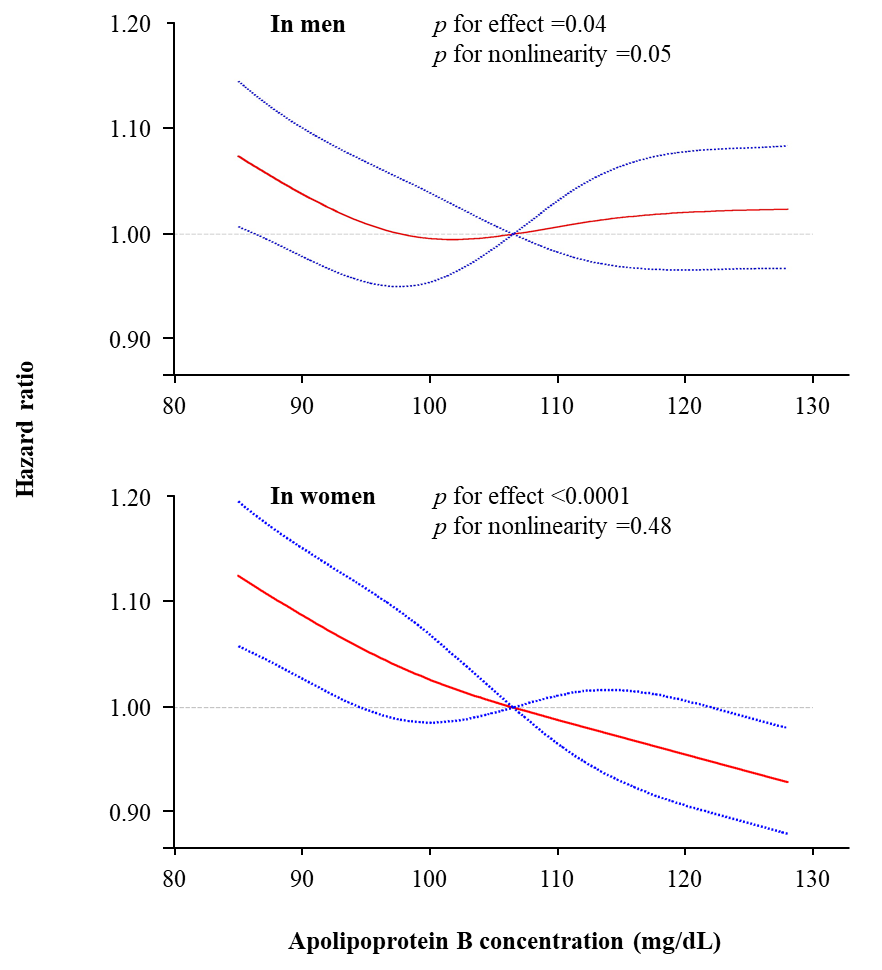


**Figure S1. Restricted cubic spline analysis of sex-specific associations between apolipoprotein B and atrial fibrillation incidence.**

**
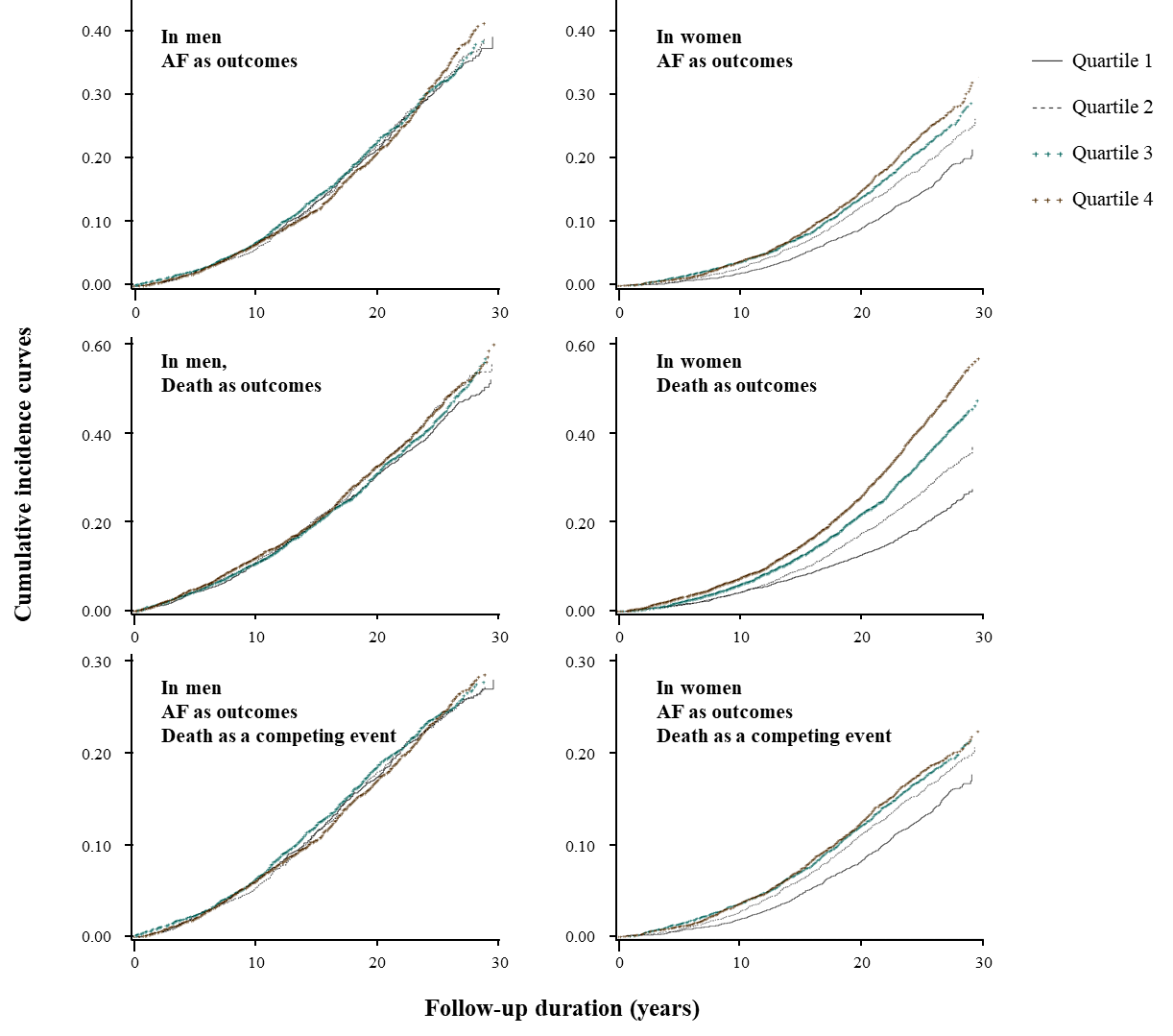
**

**Figure S2. Sex-specific cumulative incidence curves by apolipoprotein B quartiles.** AF, atrial fibrillation.
